# Homoharringtonine induced immune alteration for an Efficient Anti-tumor Response in Mouse Models of Non-small Cell Lung Adenocarcinoma Expressing Kras Mutation

**DOI:** 10.1038/s41598-018-26454-w

**Published:** 2018-05-29

**Authors:** Tzu-Yang Weng, Hsuan Franziska Wu, Chung-Yen Li, Yu-Hsuan Hung, Yu-Wei Chang, Yi-Ling Chen, Hui-Ping Hsu, Yu-Hung Chen, Chih-Yang Wang, Jang-Yang Chang, Ming-Derg Lai

**Affiliations:** 1 Department of Biochemistry and Molecular Biology, College of Medicine, National Cheng Kung University, Tainan, Taiwan; 2 Department of Surgery, National Cheng Kung University Hospital, College of Medicine, National Cheng Kung University, Tainan, Taiwan; 3 Department of Medicine, National Cheng Kung University, Tainan, Taiwan; 4 Institute of Basic Medical Sciences, College of Medicine, National Cheng Kung University, Tainan, Taiwan; 5 Department of Senior Citizen Services Management, Chia Nan University of Pharmacy and Science, Tainan, Taiwan; 6 National Institute of Cancer Research, National Health Research Institute, Tainan, Taiwan

## Abstract

Homoharringtonine (HHT), an inhibitor of protein synthesis, has been used to treat leukemia. Its therapeutic effects on non-small cell lung adenocarcinoma carrying KRAS mutation and their immune system are less understood. The present study examined the therapeutic efficacy and the immune effects of HHT in two murine lung tumor models, xenograft and transgenic, carrying the Kras mutation G12D and G12C respectively. HHT exhibited efficient anticancer activity, significantly suppressing lung tumor growth *in vitro* and *in vivo*. The levels of 22 cytokines and chemokines in splenocytes of tumor-bearing mice were examined. Interleukin-12 expression was lower in splenocytes of HHT-treated mice when compared to the controls as demonstrated by a cytokine array and an enzyme-linked immunosorbent assay. The expression levels of CD80, CD86, and CD69 in B220^+^ B cells from splenocytes of HHT-treated mice were higher than that of control mice in two mouse tumor models. Furthermore, antitumor effect of HHT was attenuated with depletion of B cells. Increased numbers of CD80^+^ and CD86^+^ B cells were observed in the mice treated with narciclasine, another translation inhibitor. In conclusion, HHT changed the features of immune cells, and exhibited efficient anti-tumor activity against lung tumor carrying mutant Kras expression.

## Introduction

Lung cancer is the leading cause of cancer-related deaths worldwide, with five-year survival rates of only 18%^1,2^. Non-small cell lung cancer (NSCLC) is responsible for approximately 80–85% of lung cancers, and it is frequently associated with genetic and epigenetic abnormalities^3^. Among somatic mutations in NSCLC, the epidermal growth factor receptor (*EGFR*) and V-Ki-ras2 Kirsten rat sarcoma viral oncogene homolog (*KRAS*) are the most commonly mutated oncogenes^2^. Several anticancer agents targeting EGFR are under development, and some have been shown to exhibit therapeutic benefits in clinical studies of NSCLC, including tyrosine kinase inhibitors and monoclonal antibodies^4,5^. In contrast, KRAS, which is the most common mutant oncogene in cancers, is observed in about 25–30% of NSCLC patients^6^. KRAS mutations usually occur in codon 12, 13, or 61 and result in constitutive activation of downstream signaling, including phosphatidyl inositol-3-kinase (PI3K)/Akt pathway or mitogen-activated protein kinase/extracellular-signal regulated kinase (MAPK/ERK) pathway^7^. Recent studies indicated that NSCLC patients with KRAS mutations were resistant to EGFR-targeted treatments, including monoclonal antibodies and tyrosine kinase inhibitors^8,9^. Potential KRAS-based treatments include MEK inhibitors, BRAF inhibitors, farnesyltransferase inhibitors, and geranylgeranyl transferase inhibitors, which target downstream signaling translocation pathways and the plasma membrane^10–13^. At present, there are no effective treatments for NSCLC patients with KRAS mutations^14^.

Dysregulation of protein translation has frequently been observed in cancers^15^, which results in abnormal cell proliferation, reduced survival, and changes in immunity^16–18^. Homoharringtonine (HHT) is a natural alkaloid isolated from*Cephalotaxusharringtonia*^19^, is used as a traditional Chinese medicine^20^. Studies conducted in China in the 1970s evidenced the therapeutic effects of HHT against chronic myelogenous leukemia (CML) and acute myelogenous leukemia (AML)^21,22^. Various clinical studies demonstrated that HHT elicited the anti-tumor activity in AML and CML, either alone or in combination with interferon-α (IFN-α) or granulocyte colony-stimulating factor^23,24^. HHT suppressed protein synthesis by inhibiting aminoacyl-tRNA binding to the A-site of ribosomes^25^, and resulted in cell apoptosis and anti-tumor effects^26–28^. HHT induced caspase-3-mediated cleavage of poly (ADP-ribose) polymerase, upregulated the expression of the pro-apoptosis factor Bax, and induced cell death in human myeloid leukemia cell lines^29^. Furthermore, the levels of Bcl-xL, p-JAK2, p-STAT5, and p-AKT were down-regulated in primary AML cells and AML cell lines following HHT treatment^30^. In a gefitinib-resistant NSCLC model, HHT induced anti-tumor effects by suppressing interleukin-6 (IL-6)/JAK1/STAT3 signaling^31^. Additionally, oncogenic RAS-activated ERK signaling was indicated that associated with the stimulations of cap-dependent translation^32^. KRAS-driven tumorigenic functions were suppressed by inhibiting the translation activity of the eIF4 complex in NSCLC *in vitro* and *in vivo*^33^.

The enhancement of anti-tumor immunity during chemotherapy in cancer patients has been identified, and correlated with clinical outcome^34^. Several ongoing clinical lung cancer trials using a combination of chemotherapy and immunotherapy are underway^35^. Some trials have revealed therapeutic benefits of targeting immune checkpoint in lung cancer^36,37^. On the other hand, cytokine production alteration by the chemotherapy or translation inhibitor may influence the immune response and the therapeutic efficacy^38,39^. The therapeutic activity of translation inhibitors against an undruggable oncogene, KRAS, in NSCLC were less addressed. Furthermore, the effects of protein translation inhibitor on the cytokine production in tumor microenvironment have not been studied yet. Thus, the present study investigated anti-tumor activity and changes in cytokine production induced by translation inhibitor, HHT, in mouse models carrying the Kras mutation. The results revealed that HHT exhibited effective anti-cancer activity in lung cancer mouse models of NSCLC and that it reduced IL-12 cytokine expression and enhanced B-cell activation.

## Results

### HHT inhibited growth of human lung tumor cell lines *in vitro*

To investigate whether HHT induced anti-tumor effects against lung adenocarcinoma, two human NSCLC cell lines (A549 and H1299) were treated with HHT, and cell proliferation was examined. HHT dramatically reduced the cell growth of A549 and H1299 cells (Fig. S1A and B) in a dose-dependent manner after 24 and 48 h. The inhibitory effects of HHT on protein expression of oncogenic-associated proteins were evaluated in NSCLC cell lines. HHT downregulated the expression of oncogenic (KRAS, ERK, Akt, STAT3, CDK4, and CDK6) and tumor suppressor proteins (p21 and RB) in the A549 cell line by western blotting (Fig. S1C).

### HHT inhibited growth of mouse lung tumor cells carrying a Kras mutation *in vivo*

**Figure 1:**
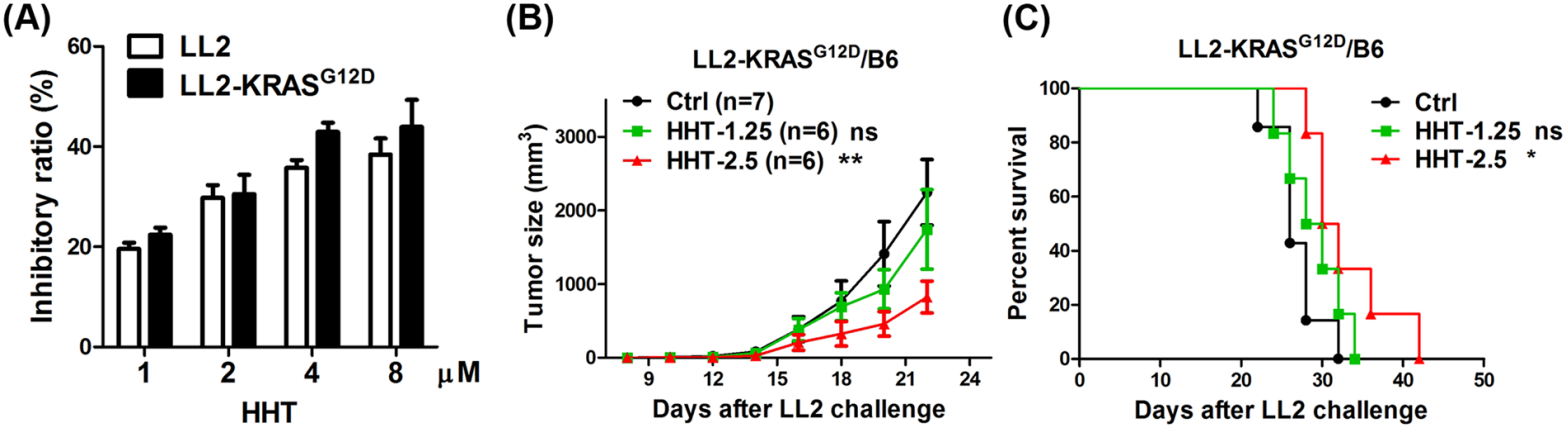
HHT suppressed lung tumor growth in tumor-bearing mice carrying a Kras mutation. (**A**) HHT-treated LL2 cells carrying the KRAS^G12D^ mutation, as measured by the WST-1 assay. The cells were treated with 1, 2, 4, and 8 μM HHT for 48 h. The cell viability was measured using a WST-1 assay. Three independent experiments were performed. (**B**) Kras^G12D^-expressing LL2 tumor-bearing mice were treated with 1.25 or 2.5 mg/kg HHT, and the tumor volume was measured at the indicated times. (**C**) Survival of Kras-mutant carried LL2 tumor-bearing mice using the Kaplan-Meier analysis. *p < 0.05, **p < 0.005.ns, no statistical difference.

To evaluate the therapeutic effects of HHT in lung cancer cells expressing a Kras mutation, mouse LL2 cells were transduced with lentivirus carrying mouse Kras^G12D^. Expression of exogenous Kras^G12D^ was demonstrated by immunoblotting (Fig. S2A). The cell growth of Kras^G12D^-expressing LL2 was examined. HHT exhibited suppressive effect on parental LL2 cell lines and LL2 cell lines expressing mutant Kras (Fig. 1A). Protein inhibition were observed in LL2 cells, in which the expression of endogenous ERK, STAT3, and Akt was reduced (Fig. S2B).

HHT inhibited the cell growth in Kras^G12D^-expressing LL2 cell lines. In order to investigate the therapeutic effects of HHT *in vivo*, Kras^G12D^-expressing LL2 tumor-bearing mice were treated with HHT (1.25 mg/kg and 2.5 mg/kg) by intraperitoneal (i.p.) injections. The tumoral volume of the Kras^G12D^-expressing LL2 was inhibited by the HHT (2.5 mg/kg) treatment (Fig. 1B). The lifespan of the HHT-treated tumor-bearing mice with Kras^G12D^-expressing LL2 was prolonged when compared to control mice (Fig. 1C). It was interesting to note that HHT exerted similar inhibitory effects on the parental LLC and LLC expressing active Kras (Fig. 1A), indicating that the effects of HHT is not specific to Ras signal pathway.

### HHT altered the cytokine expression in immune cells through suppressing the protein synthesis

**Figure 2:**
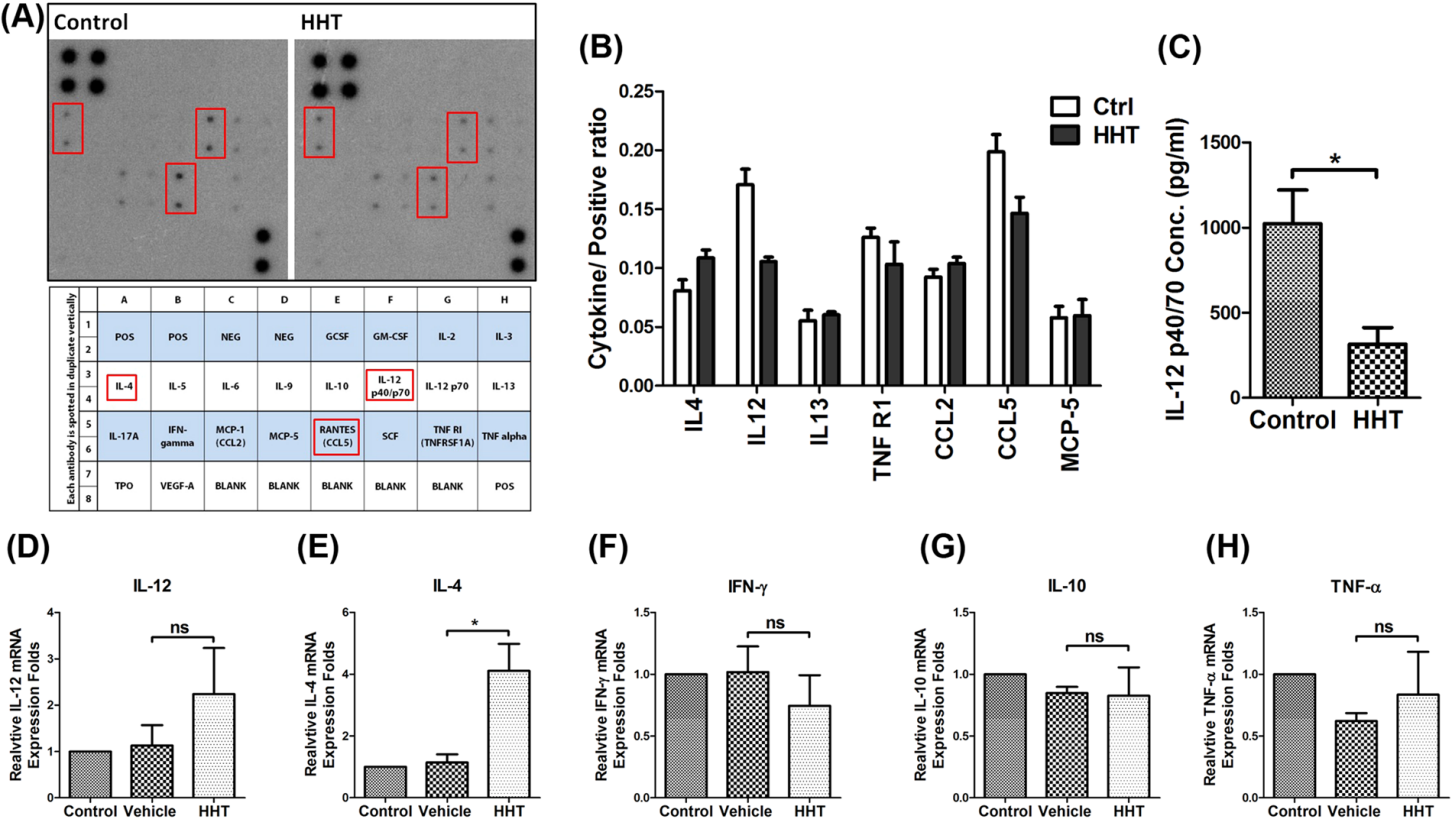
HHT altered the protein expression in splenocytes from LL2-tumor mice carrying a Kras mutation. (**A**) *Upper panel*, showing cytokine expression levels in culture media of splenocytes from Kras^G12D^-expressing LL2 tumor-bearing mice treated with HHT, detected by a cytokine antibody array. *Lower panel*, showing an array map. (**B**) Expression levels of cytokines normalized to those of a positive control. Data are shown for two independent experiments. (**C**) IL-12 expression levels of splenocytes isolated from Kras^G12D^-expressing LL2 tumor-bearing mice were measured using an ELISA. The columns and bars represent mean values ± SEM (*n* = 3 per group). The mRNA expression levels of (**D**) IL-12, (**E**) IL-4 (**F**) IFN-γ, (**G**) IL-10 and (**H**) TNF-α were measured by the real-time PCR method. The bar graphs represent the quantification of cytokine expression in the spleen. The relative gene expression of each group was normalized to that of splenocytes from control mice that received no treatment. The columns and bars represent mean values ± SEM (*n* = 3 per group). *p < 0.05. ns, no statistical difference.

HHT is thought to be a global translation inhibitor. To investigate the effects of HHT on immune response, the levels of 22 cytokines and chemokines from splenocytes of Kras^G12D^-expressing LL2 tumor-bearing mice treated with HHT were measured using a cytokine array. A number of cytokine levels were altered by the treatment of HHT (Fig. 2A). Among those cytokines, the splenic secretion of IL-12 (p40/p70) was consistently decreased in the HHT-treated mice as compared to the control mice (Fig. 2B). Splenic secretion of IL-12 was further quantified by an enzyme-linked immunosorbent assay (ELISA). The IL-12 levels in culture medium of splenocytes of the HHT-treated groups exhibited a two-fold decrease compared to those of the control groups (Fig. 2C). Next, the mRNA expression of cytokines was evaluated to explore whether the HHT-induced changes in cytokine expression were due to the suppression of protein synthesis or alternations in mRNA levels. The mRNA levels of IL-12 in the HHT groups were slightly enhanced compared with those of the vehicle control, but the difference was not statistically significant (Fig. 2D). The mRNA levels of IL-4 in the HHT groups were higher than those of the control group (Fig. 2E). The changes in IL-4 protein and IL-4 mRNA levels were more apparent in the HHT groups than in the control groups (1.2-fold higher in protein levels; 4-fold higher in mRNA levels) (Fig. 2B and E). There were no significant differences in IL-10, interferon-γ (IFN-γ), and tumor necrosis factor-α (TNF-α) expression in the splenocytes of the vehicle and HHT groups (Fig. 2F–H). These results suggested that HHT induced changes in cytokine expression mainly via the suppression of protein synthesis, not mRNA transcription. HHT-induced dysregulation of protein expression in immune cells, including the suppression of IL-12, indicating that HHT may potentially interfere with immune homeostasis.

### HHT treatment altered the properties of B cells in mice bearing Kras^G12D^-expressing LL2 tumor cells

**Figure 3:**
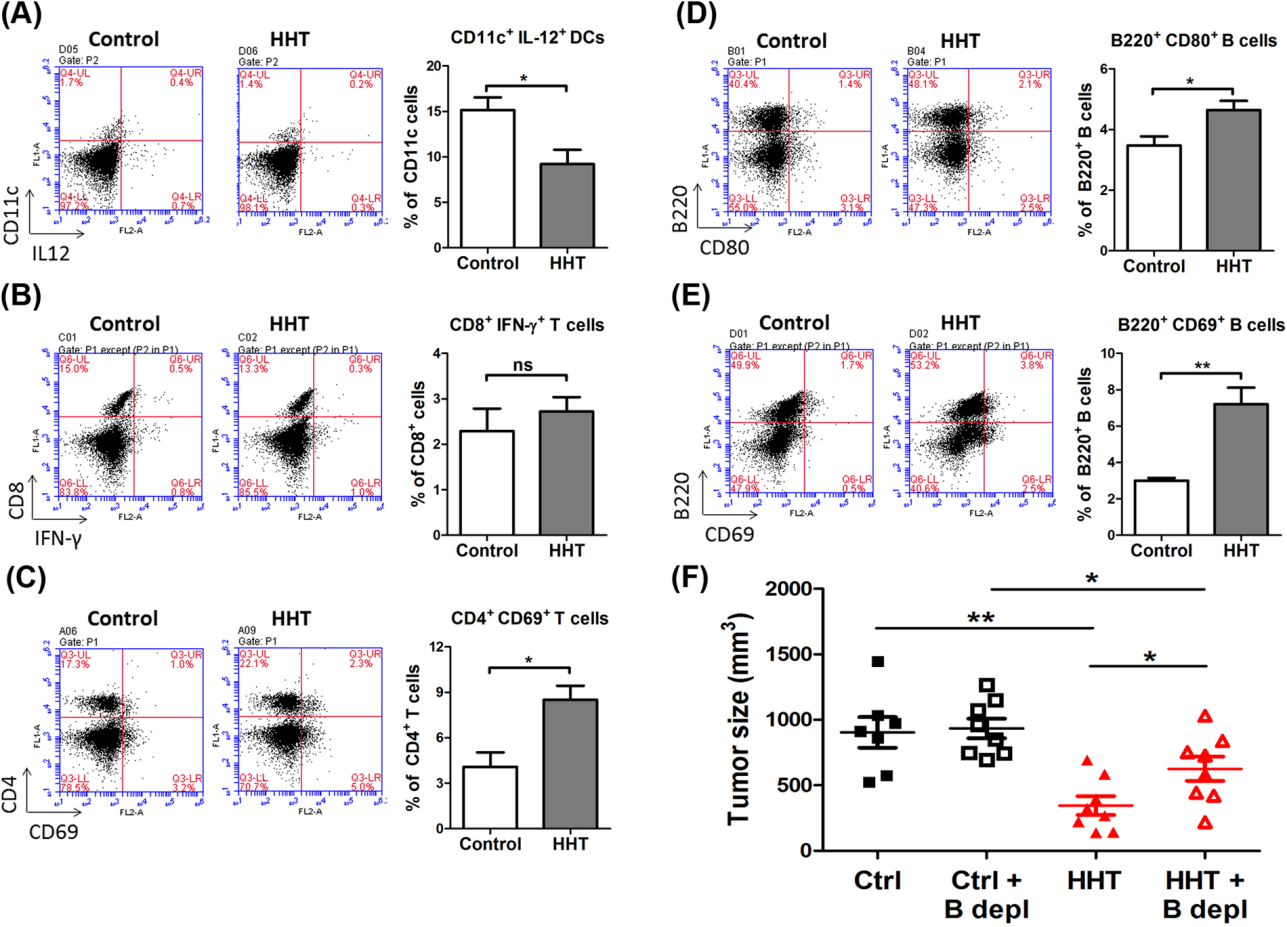
HHT treatment influenced characteristics of splenocytes. Splenocyteswere isolated from Kras^G12D^-carrying LL2 tumor-bearing mice, which were treated with HHT (2.5 mg/kg) at the indicated days. (**A**) FACS analysis of CD11c^+^ IL12^+^ dendritic cells (*n* = 4 mice per group). (**B**) FACS analysis of CD8^+^IFN-γ^+^ T cells (*n* = 3 mice per group). (**C**) FACS analysis of CD4^+^ CD69^+^ T cells (*n* = 4 mice per group). (**D**) FACS analysis of B220^+^ CD69^+^ B cells (*n* = 3 mice per group). (**E**) FACS analysis of B220^+^ CD80^+^ B cells (*n* = 4 mice per group). The bar graph represents the average ± SEM. (**F**) Kras^G12D^-expressing LL2 tumor-bearing mice with B-cell depletion and treated with HHT. The tumor volume was measured on day 23. Ctrl, control. B depl, B-cell depletion. *p < 0.05 and **p < 0.005. ns, no statistical difference.

IL-12, a major cytokine secreted by splenic dendritic cells (DCs), enhanced Th1-immunity^40^; therefore, we examined the expression of IL-12 in CD11c^+^ DCs by flow cytometry. The effects of HHT-suppressed IL-12 were analyzed by measuring the expression of activated immune cells, including CD8^+^ T cells, CD4^+^ T cells, and B220^+^ (CD45R^+^) B cells. The production of IL-12 in CD11c^+^ DCs and other splenic cells was significantly decreased in the HHT-treated mice compared with control mice (Fig. 3A). There was no difference in IFN-γ expressing CD8^+^ T cells between the HHT and control groups (Fig. 3B). In contrast, the expression of CD69, a marker of early activation, was significantly increased in CD4^+^ T cells of the HHT-treated mice compared with those of the controls (Fig. 3C). As reported previously, high levels of B7-1/2 (CD80/CD86) and CD69 in murine B cells served as markers of activated B-cell subsets^41–43^. The expression levels of CD80 and CD69 in B220^+^ B cells in the splenocytes of the HHT-treated mice were elevated (1.4-fold and 2-fold, respectively) compared to those of the controls, suggesting that HHT enhanced the activation of B cells (Fig. 3D and E). In order to evaluate the significance of B cells in the anti-tumor response induced by HHT, the therapeutic effects of HHT were examined by the depletion of B cells *in vivo*. In Kras^G12D^-expressing tumor-bearing mice, therapeutic efficacy of HHT was attenuated by the depletion of B cells when compared with control mice without depletion (Fig. 3F). Altogether, the changes in the properties of immune B cells by HHT contributed to part of the anti-tumor effects of HHT in the KRAS^G12D^-expressing mouse tumor model. HHT may be a potent therapeutic drug against lung cancer associated with KRAS mutations through direct killing of cancer cells and indirect influencing immune microenvironments.

### Administration of recombinant IL-12 altered HHT-induced anti-tumor effects and immune response in Kras^G12D^-expressing LL2 tumor bearing mice

To investigate the role of IL-12 in HHT-induced immune modulation *in vivo*, tumor-bearing mice were treated with HHT plus high (0.5 μg/mice) or low dose (0.05 μg/mice) of recombinant IL-12 (0.05 μg or 0.5 μg/mice). Low dose of recombinant IL-12 attenuated the anti-tumor effect of HHT (Fig. S3A). In addition, low dose of recombinant IL-12 (0.05 μg) significantly decreased the population of B220^+^ CD86^+^ B cells (Fig. S3B). Furthermore, low dose of recombinant IL-12 decreased the amount of CD4^+^ CD69^+^ T cells, though not statistically significant (Fig. S3C). In contrast, combination of high dose of recombinant IL-12 (0.5 μg) and HHT increased the cancer therapy effects (Fig. S3D). These results suggested that decrease of IL-12 may be responsible for the increase of B220^+^ CD86^+^ B cells during HHT treatment (Figs 2 and 3). It was interesting to note that high dose of IL-12 can further enhance HHT-induced therapeutic effects, indicating the influence of IL-12 is dose-dependent.

### The effects of HHT on tumor growth and B-cell activation in a Kras^G12C^-driven lung tumor transgenic mice

**Figure 4:**
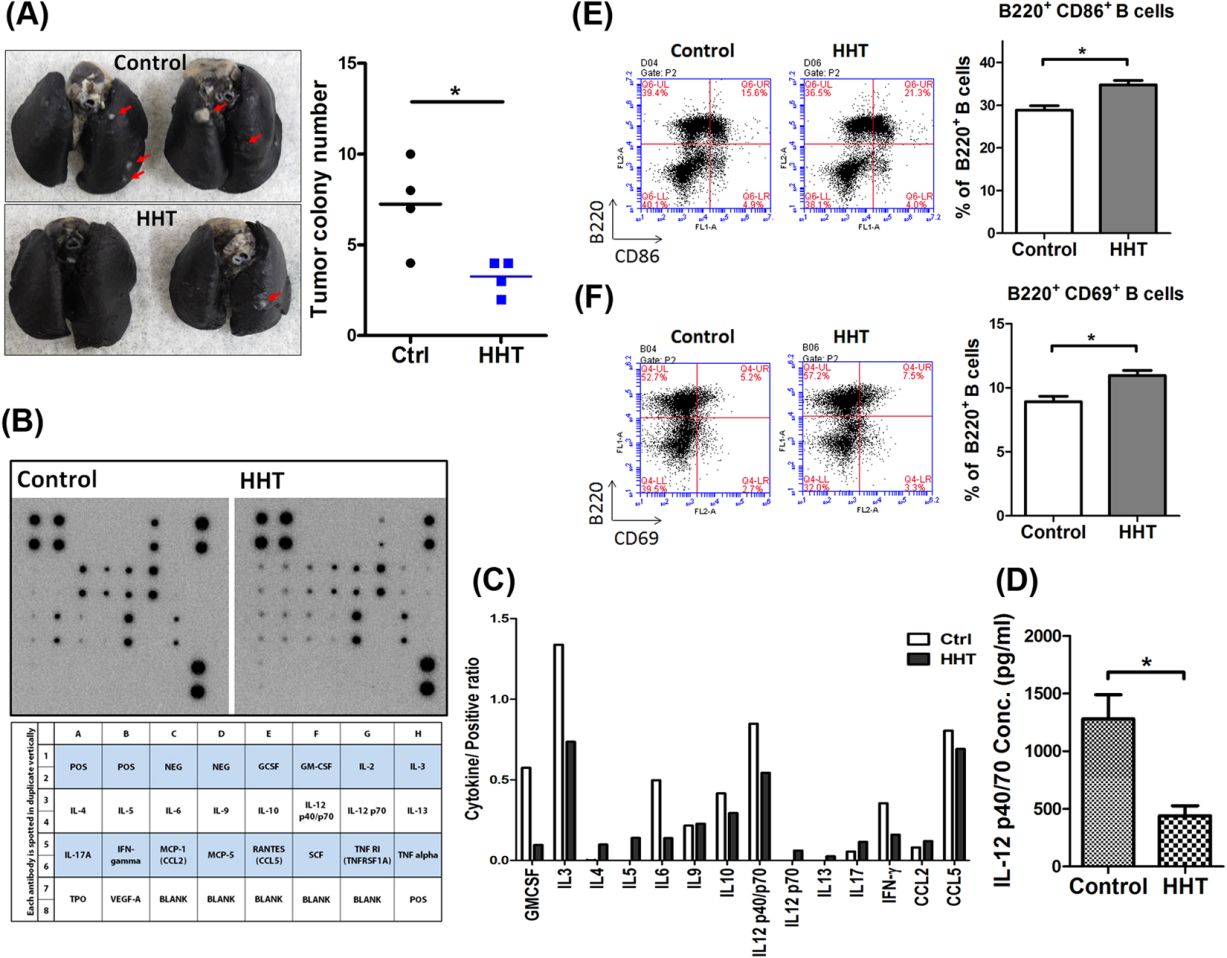
HHT induced anti-tumor effects and alteration of immune cells in a Kras^G12C^-driven spontaneous mouse lung tumor model. (**A**) *Left panel*, India ink staining of lung tumors from the mice treated with and without HHT. *Right panel*, showing the number of lung tumor nodules. (**B**) *Upper panel*, showing cytokine expression levels in culture media of splenocytes from Kras^G12C^ bi-transgenic mice treated with HHT, as detected by a cytokine antibody array. *Lower panel*, showing an array map. (**C**) Cytokine expression levels normalized to those of a positive control. (**D**) IL-12 expression levels of splenocytes isolated from Kras^G12C^-expressing tumor-bearing mice were measured using an ELISA. The columns and bars represent mean values ± SEM (*n* = 3 per group). (**E**) FACS analysis of B220^+^ CD86^+^ B cells and (**F**) B220^+^ CD69^+^ B cells in the spleen. The bar graph represents the average ± SEM (*n* = 3 mice per group). *p < 0.05, **p < 0.005, and ***p < 0.001. ns, no statistical difference.

We further investigated the therapeutic effects and changes in immune cells induced by HHT in a genetically engineered inducible Kras^G12C^-driven lung tumor transgenic mouse model^44^. The numbers of tumor nodules were significantly decreased in the HHT-treated mice compared with the mice that received no HHT treatment (Fig. 4A). The cytokine expression profiles and properties of immune cells were evaluated using a cytokine array assay and flow cytometry analysis. The expression of various cytokines, including IL-3, IL-6, IL-12 p40/p70, IFN-γ, and granulocyte-macrophage colony-stimulating factor, was dramatically suppressed in the splenocytes of the HHT-treated mice compared with splenic expression in the KRAS^G12C^ transgenic mouse model (Fig. 4B and C). The quantitative results of the ELISA analysis revealed that the IL-12 p40/p70 level was decreased more than two-fold in the splenocytes of the HHT group as compared with the control groups in the Kras^G12C^-driven lung tumor transgenic mice (Fig. 4D). Similar results were observed in the splenocytes of the Kras^G12C^-expressing LL2 tumor-bearing HHT-treated mice, with a consistent reduction (approximately 50%) in IL-12 levels in the HHT group compared with the control group (Fig. S4A). Altogether, the level of IL-12 was decreased in both the subcutaneous and transgenic lung tumor models, irrespective of the mutation status G12C or G12D (Figs 2C, 4D and S4B). The expression of CD69, CD80, and CD86 was also increased on surface of B220^+^ B cells in the splenocytes of the HHT-treated mice compared with the control mice (Figs 4E,F and S5A). There were no significant changes in the expression of CD4^+^ CD69^+^ T cells in the control versus the HHT groups (Fig. S5B). These data indicated that HHT is an effective therapeutic agent in lung cancer mouse models with a Kras^G12C^ mutation and that the therapeutic effects may be regulated in part by the suppression of cytokines, such as IL-12.

### Elongation-targeted translation inhibition led to polarization of the immune response to Th2 cells

**Figure 5:**
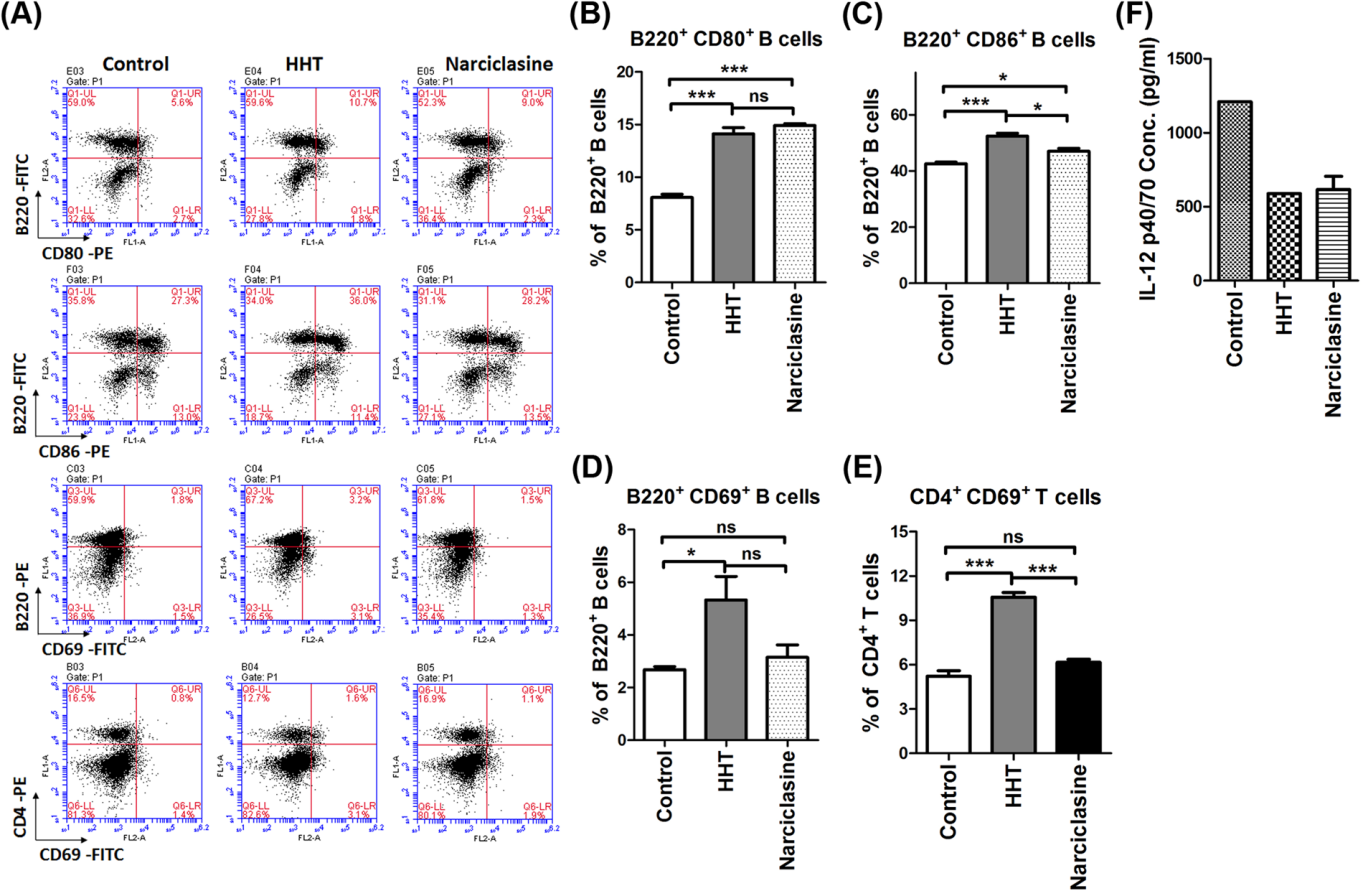
Translation inhibitors repressed IL-12 and enhanced the activation of B cells in LL2 tumor models. (**A**) Representative FACS plots of activated B and T cells in the spleens of Kras^G12D^- expressing LL2 tumor-bearing mice treated with HHT or narciclasine. (**B**) The average percentage of B220^+^ CD80^+^ B cells. (**C**) The average percentage of B220^+^ CD86^+^ B cells. (**D**) The average percentage of B220^+^ CD69^+^ B cells. (**E**) The average percentage of CD4^+^ CD69^+^ T cells. The bar graph represents the average ± SEM (*n* = 3 mice per group). (**F**) IL-12 expression levels of splenocytes isolated from KRAS^G12D^-expressing tumor-bearing mice were measured using an ELISA. Mice were treated with HHT or narciclasine. The columns and bars represent mean values ± SEM (*n* = 2 in NAR group). *p < 0.05, ***p < 0.001. ns, no statistical difference.

HHT is reported to suppress protein synthesis by occupying the elongation site of ribosomes^45^. To evaluate whether the HHT-triggered immune response to Th2 can be observed in other translation inhibitors, the effect of a 60 S inhibitor, narciclasine (NAR)^46^, was studied in the Kras^G12D^-LL2 tumor-bearing mouse model. The tumor-bearing mice treated with HHT and NAR exhibited increased CD80 and CD86 expression in B220^+^ B cells of splenocytes as compared to untreated mice (Fig. 5A,B and C). The expression of CD69 in both T cells and B cells are not statistically different between the control and NAR groups (Fig. 5A,D and E). The expression of IL-12 p40/p70 was downregulated in splenocytes of both the NAR-treated mice and HHT-treated mice compared with those of control mice (Fig. 5F). Taken together, these data indicated that the protein synthesis inhibitors HHT and NAR suppressed the protein level of IL-12 and modulated the properties of B cells *in vivo*.

## Discussion

KRAS mutations are common in various types of cancer, and it has been studied as a drug target for many years. However, few KRAS-specific drugs have thus far demonstrated significant benefits in clinical trials. In the present study, HHT exhibited anti-tumor activity in xenograft mouse tumor models carrying a Kras mutation Kras^G12D^ and in a transgenic mouse lung tumor model induced by Kras^G12C^. These results point to the clinical potential of HHT as a treatment for NSCLC patients with Kras mutations. Additionally, the cytokine expression patterns have been reported to be associated with clinical outcome in cancer patients^47^. IFN-γ and IL-2 predict a good prognosis; while serum level of IL-10 are correlated with worse prognosis in more than ten types of cancers^47^. Furthermore, IL-12 is one of the key cytokines that promoted Th1 responses, and induces the expression of IFN-γ from T cells and NK cells. Therefore, the downregulation of IL-12 may impair the activity of effector cells which results in the poor anti-tumor immunity^40^. Our present work indicated that decreasing IL-12 expression induced by HHT results in immunity toward Th2 against tumor, suggested that humoral immunity may play an anti-cancer role in these mouse lung tumor models.

Enhancing cytotoxic T-cell activity and antibody-dependent cellular cytotoxicity activity to induce the death of tumor cells is considered to be an efficient approach in cancer immunotherapy^48^. On the other hand, there is no consensus on the potential roles of Th2 or B cells in tumor development. Previous research reported that B cells promoted tumor progression and tumorigenesis by enhancing angiogenesis, thereby creating a pro-inflammatory microenvironment and suppressing T-cell activation^49^. For example, IL-6 binding to CD5^+^ B cells promoted tumor growth by activating STAT3 in mouse tumor models, and were observed to be correlated with phosphorylated STAT3 in multiple human tumors, including NSCLC^50^. B-cell-producing cytokines were shown to induce chronic inflammation associated with the development of spontaneous cancers in mice^51^.

In contrast, anti-tumor effects have been reported to be associated with B cells.The presence of tertiary lymphoid structures, similar to ectopic lymphoid organs, in lung tumors appeared to be associated with a better prognosis in patients and to be associated with local anti-tumor B cell–mediated immunity^52^. B cells also play a role as antigen-presenting cells, generating tumor-associated antigen-specific CD8^+^ and CD4^+^ T cells^53,54^, pointing to crosstalk between B and T cells in anti-tumor immunity.

HHT decreased the STAT3 protein levels (Figs S1C and S2B). STAT3 plays an important role in oncogenesis and immune suppression. The function of STAT3 can be either oncogene or tumor suppressor depending on the mutation status of cancer cells^55^. Mutant EGFR promotes the secretion of the cytokines, IL-6^56^ and IL-10, which induced STAT3 activation in immune cells, thereby suppressed the immune function^57^. STAT3 inhibited the activation of GATA3 and STAT6 through miR-135b in anaplastic large cell lymphoma^58^. GATA3 played an important switch in regulating Th1/Th2 cell differentiation. GATA3 induced naïve CD4^+^ T cells into Th2 cells and increased the IL-4 expression^59^. Moreover, production of IL-4 induced proliferation, differentiation and activation of B cells^60^. We speculate that STAT3 may be one of the important factors contributing to activation of B cells and increased IL-4 mRNA level through GATA3. Our results also indicated that HHT-induced therapeutic effects was attenuated by B-cell depletion, indicating that HHT promoted B-cell activation and potentially resulted in anti-tumor activity. Therefore, downregulation of STAT3 may be essential for HHT-mediated anti-cancer therapy.

Previous efforts targeting KRAS efforts focused on the following therapeutic strategies: blocking KRAS translocation, directly attacking the G domain, targeting the downstream of KRAS, and suppressing KRASexpression^61^. Among these, the PI3K/Akt pathway and MAPK/ERK pathway are the well-known downstream of KRAS in lung tumors. AKT or STAT3 activation contributed to the effects of the MEK inhibitor in lung cancer cells with a KRASmutation^62^. CDK4 played a major role in KRAS -induced tumorigenesis^63^. Additionally, Ras/PI3K and Ras/Erk/MAP kinase-activated protein kinase (Mnk)1 and 2 increased translation throughmediating the eukaryotic initiation factor eIF4E, which is essential for the translation regulation of cellular processes, including cell growth and proliferation^64,65^. In the present work, HHT markedly suppressed the expression of oncogenic proteins (Kras, ERK, Akt, STAT3, CDK4, and CDK6) and tumor suppressors (p21 and RB) in NSCLC cell lines. By disrupting the expression of many proteins, tumoral cell growth was inhibited in NSCLC cells in *vitro* and in *vivo*.

Various functions of adaptive and innate immunity have been identified, which could be modulated through the control of translation mechanism^39^. On the other hand, dysregulation of protein synthesis has been observed in several cancers. Chemotherapeutic drugs targeting ribosome biogenesis have attracted much attention as anti-cancer treatments. To the best of our knowledge, this is the first study to investigate the anti-cancer efficacy of HHT, and the effects of translation inhibitors on immune alteration for an efficient anti-tumor response. The findings of this study may contribute to combinational immunotherapy involving translation inhibitors in the clinical setting.

## Material and Methods

### Cell lines and reagents

Human lung cancer cell lines were kind gifts from Professor Pan-Chyr Yang (National Taiwan University, Taipei, Taiwan)^66^. The LL2 murine lung cancer cell line was a kind gift from Professor Chao-Liang Wu. The LL2and A549 cells were grown in Dulbecco’s modified Eagle medium (Gibco, Carlsbad, CA, USA). Both culture media were supplemented with 10% fetal bovine serum (FBS) (Gibco), 100 U/ml of penicillin, and 100 mg/ml of streptomycin (Hyclone, Logan, UT, USA). The cells were maintained at 37 °C in a 5% CO_2_ incubator. HHT was purchased from Sigma-Aldrich (H0635; St. Louis, MO, USA) and Toronto Research Chemicals (H596500; Toronto, Ontario, Canada). NAR was purchased from Cayman Chemical (20361; Ann Arbor, MI, USA). Both compounds were dissolved in ethanol (2 mM or 50 mg/ml) and diluted in phosphate buffered saline (PBS), 2 µM for *in vitro* experiments and 2.5 mg/ml for animal experiments. Recombinant mouse IL-12 was purchased from BioLegend (577006; San Diego, CA, USA). Recombinant IL-12 was diluted in PBS (2.5 or 0.25 mg/ml) for animal experiments.

### Cell proliferation assay

Cell proliferation was examined using a WST-1 cell proliferation reagent (Clontech, Mountain View, CA, USA). The WST-1 assay was performed in accordance with the manufacturer’sprotocol, with little modification. A549 human lung cancer cells were transfected with a pcDNA3.1 KRAS^G12D^ plasmid for 24 h and then seeded in 96-well plates at a cell density of 5,000 cells per well. After 12 h, HHT was added to each well at the recommended concentration for 24 or 48 h. LL2 mouse lung cancer cells were infected with a lentivirus carrying Kras^G12D^ plasmid for 48 h and then seeded in 96-well plates at a cell density of 5,000 cells per well. After 12 h, HHT was added to each well at the recommended concentration for 48 h. WST-1 reagent (10 µL/well) was added to the culture wells and incubated for 1 h. Absorbance was measured at a wavelength of 450 nm using a scanning multi-well spectrophotometer.

### Western blot analysis

The following antibodies were used in Western blotting: anti-β-actin (GTX110564; GeneTex, Hsinchu, Taiwan), anti-Kras (F234) (sc-30; Santa Cruz Biotechnology, CA, USA), anti-ERK (pan ERK) (610123; BD Pharmingen, San Diego, CA, USA), anti-AKT (H136; Santa Cruz Biotechnology), anti-Stat3 (610189;BD Pharmingen), anti-CDK4 (ab108355; Abcam, Cambridge, UK), anti-CDK6 (ab124821; Abcam), anti-p21 (GTX63148; GeneTex, Hsinchu, Taiwan), and anti-RB (554136; BDPharmingen). To examine expression efficiency of KRAS^G12D^, the A549 cells were transfected with 1 μg of human KRAS^G12D^ plasmid. The LL2 cells were infected with a lentivirus carrying Kras^G12D^ for 48 h. HHT (2 µM) was then added to the cells for 24 h. Cell lysates were prepared by treating the cells with RIPA lysis buffer (0.22 M NaCl, 0.38 M Tris-HCl, pH 7.5, 0.25% sodium deoxycholate, and 1% IGEPAL-630). The protein concentration was measured using a Micro BCA™ protein assay reagent kit (Pierce, Rockford, IL, USA). Polyvinylidene fluoride membranes were incubated overnight at 4 °C with the primary antibody in TTBS containing 1% bovine serum albumin. The secondary antibody was subsequently incubated with the membranes for 1 h at room temperature. The membranes were then washed extensively for 30 min with TTBS at room temperature. The blots were probed with an ECL Western blot detection system and visualized with the BioSpectrum AC imaging system (UVP, CA, USA), according to the manufacturer’s instructions.

### Animal tumor models

All experiments in this study involving mice were approved by the Institutional Animal Care and Use Committee of National Cheng Kung University (approval no. NCKU-IACUC-103-231). The methods were performed in accordance with the approved guidelines. Female C57BL/6 mice aged six to eight weeks were obtained from the Laboratory Animal Center at National Cheng Kung University (Tainan, Taiwan). LL2 cells (2 × 10^5^ cells in 200 μl of PBS) were injected via the subcutaneous (s.c.) route into C57/BL6 mice. Tumor-bearing mice received intraperitoneal (i.p.) injections of HHT (1.25–2.5 mg/kg) on day 10 after the tumor challenge, at two-day intervals, with a total of 10 i.p. injections administered. Tumor-bearing mice received intraperitoneal (i.p.) injections of HHT (2.5 mg/kg) on day 10 after the tumor challenge, at two-day intervals, and injection of IL-12 (0.05 microgram each time) after 1 day of HHT injection with a total of 3 i.p. injections administered. The tumor volume was measured using calipers and was calculated using the following formula: volume = (A^2^ × B × 0.5236), where A and B represented the shortest and longest diameters, respectively. The mice were sacrificed when the tumor volume exceeded 2,500 mm^3^ or when they were expected to shortly become moribund.

FVB.Cg-Tg(Scgb1a1-rtTA)1Jaw/J transgenic mice (006222) were obtained from the laboratory of Professor Jan-Jong Hung and maintained at the National Laboratory Animal Center in Taiwan. FVBTg(tetO/CMVKRAS*G12C)9.1Msmi/J transgenic mice (006439) were acquired from the Jackson Laboratory (Bar Harbor, MA, USA). After genotyping, six-week-old bi-transgenic mice were treated with doxycycline (0.4 g/ml) in drinking water to induce tumor formation until sacrificed. For therapeutic experiments, the transgenic mice were treated with HHT (1.25 or 2.5 mg/kg) on the day after tumor induction for eight weeks, at four-day intervals, with a total of 20 HHT injections administered. Two weeks after the final treatments, the mice were sacrificed to evaluate the therapeutic effects of the indicated treatments. Lungs were excised, injected with India ink, and fixed in Fekete’s solution for counting of tumor nodules^67^.

### B-cell depletion

The protocol for *in vivo* B cell depletion was as described in a previous study^68^, with little modification. Anti-CD19 (1D3) (BioXcell, West Lebanon, NH, USA) (300 μg per mouse), anti-B220 (RA3.3A1/6.1) (BioXcell) (300 μg per mouse), or a rat IgG2a isotype control antibody (BD Pharmingen) was injected via the i.p route into mice on days 7, 8, 15, and 22. About 90% of B220^+^ B cells were depleted as determined by flow cytometry analysis.

### Flow cytometry analysis

Splenocytes were collected from the spleens of the mice one day after the third HHT or NAR treatment and filtered through a 0.7-μm cell strainer (BD Pharmingen). To stimulate an immune response, the splenocytes were incubated with LL2-KRAS^G12D^ or LL2-KRAS^G12C^ cell lysates. The following antibodies were used in the flow cytometry analysis: FITC-conjugated anti-CD4 (557307; BD Pharmingen), FITC-conjugated anti-CD8a (553031; BD Pharmingen), FITC-conjugated anti-CD45R (103206; Biolegend, San Diego, CA, USA), FITC-conjugated anti-CD45R (561878; BD Pharmingen), PE-conjugated anti-CD69 (12-0691-81; eBioscience, San Diego, CA, USA), PE-conjugated anti-CD80 (561955; BD Pharmingen), PE-conjugated anti-CD86 (561878; BD Pharmingen), FITC-conjugated anti-CD86 (561962; BD Pharmingen), PE-conjugated anti-IL-12 (526035; BD Pharmingen), PE-conjugated anti-Foxp3 (12-5773-80; eBioscience), and PE-conjugated anti-IFN-γ (BD Pharmingen) antibodies. A Transcription Factor Buffer Set (BD Pharmingen™) was used for intracellular staining of IL-12, Foxp3, and IFN-γ. All surface and intracellular staining protocols were performed in accordance with the instructions of the manufacturers. A BD Accuri C6 system (BD Pharmingen) was used to determine protein expression. To analyze the percentage of specific subpopulations of B cells, DCs, or T cells in the spleen, the immune cells were gated based on the side- and forward-scatter characteristics of the T cells.

### Measurement of cytokine expression and IL-12 p40/70

To analyze cytokine expression, splenocytes were collected from the spleens of tumor-bearing mice or bi-transgenic mice one day after the HHT treatments. The splenocytes (5 × 10^6^) were incubated with LL2 tumor cell lysate with mutant Kras expression for 24 h in 2 ml of RPMI1640 media (10% FBS) in six-well dishes, followed by incubation in 2 ml of FBS-free RPMI1640 media without tumor cell lysate for 48 h. The culture media were collected and centrifuged for 5 min at 1,500 g. Culture supernatant were stored at −80 °C until used. A mouse cytokine C1 array (RayBiotech, Norcross, GA, USA) was then used to determine the expression levels of 22 cytokines or chemokines in the culture media.

For cytokine array assays, 1 ml of undiluted sample was incubated overnight with antibody arrays at 4 °C. The membranes were incubated with a biotinylated antibody cocktail overnight at 4 °C and then incubated with HRP-streptavidin for 2 h at room temperature. The antibody array membranes were exposed to X-ray film, and protein quantitation was done using ImageJ software. The level of IL-12 p40/70 was measured using ELISA kits (ELM-IL12P40P70; RayBiotech). All the experiments were performed in accordance with the manufacturer’s instructions.

### Real-time PCR

Splenocytes were isolated from tumor-bearing mice or bi-transgenic mice after the third treatments with PBS, HHT, or NAR and incubated for one day with tumor cell lysate expressing mutant Kras. The following HPRT, IL-4, IL-10, IL-12, IFN-γ, and TNF-α sense and antisense primers were used, as previously described^61^ (Supplementary Table S1). HPRT served as an internal control. Real-time PCR was performed on a StepOne™ real-time PCR instrument (Applied Biosystems, Foster City, CA, USA), using Fast SYBR Green Master Mix (Applied Biosystems). The cycling conditions were 10 min at 95 °C and 45 cycles at 95 °C for 15 s and 60 °C for 60 s. The 2^ΔΔCt^ method was used to calculate the relative RNA expression, which was normalized to HPRT expression.

### Statistical analysis

All statistical analyses were performed using GraphPad Prism 5 software. The student’s *t*-test was used to analyze the difference between groups, including differences in tumor nodule counts, mRNA expression levels, and cell percentages. A two-way ANOVA test was performed for the statistical analysis of tumor size. Kaplan–Meier analysis was performed to determine the survival rates of the mice. *P* values < 0.05 were considered statistically significant.

## Electronic supplementary material

Supplementary Figures

## References

[1] Siegel R, Ma J, Zou Z, Jemal A (2014). Cancer statistics, 2014. CA: a cancer journal for clinicians.

[2] Zappa C, Mousa SA (2016). Non-small cell lung cancer: current treatment and future advances. Translational lung cancer research.

[3] Herbst RS, Heymach JV, Lippman SM (2008). Lung cancer. The New England journal of medicine.

[4] Maemondo M (2010). Gefitinib or chemotherapy for non-small-cell lung cancer with mutated EGFR. The New England journal of medicine.

[5] Pirker R, Filipits M (2011). Monoclonal antibodies against EGFR in non-small cell lung cancer. Critical reviews in oncology/hematology.

[6] Roberts PJ, Stinchcombe TE (2013). KRAS mutation: should we test for it, and does it matter?. Journal of clinical oncology: official journal of the American Society of Clinical Oncology.

[7] Kranenburg O (2005). The KRAS oncogene: past, present, and future. Biochimica et biophysicaacta.

[8] Pao W (2005). KRAS mutations and primary resistance of lung adenocarcinomas to gefitinib or erlotinib. PLoS medicine.

[9] Eberhard DA (2005). Mutations in the epidermal growth factor receptor and in KRAS are predictive and prognostic indicators in patients with non-small-cell lung cancer treated with chemotherapy alone and in combination with erlotinib. Journal of clinical oncology: official journal of the American Society of Clinical Oncology.

[10] Davies BR (2007). AZD6244 (ARRY-142886), a potent inhibitor of mitogen-activated protein kinase/extracellular signal-regulated kinase kinase 1/2 kinases: mechanism of action *in vivo*, pharmacokinetic/pharmacodynamic relationship, and potential for combination in preclinical models. Molecular cancer therapeutics.

[11] Gilmartin AG (2011). GSK1120212 (JTP-74057) is an inhibitor of MEK activity and activation with favorable pharmacokinetic properties for sustained in vivo pathway inhibition. Clinical cancer research: an official journal of the American Association for Cancer Research.

[12] Sebti SM, Hamilton AD (2000). Farnesyltransferase and geranylgeranyltransferase I inhibitors and cancer therapy: lessons from mechanism and bench-to-bedside translational studies. Oncogene.

[13] Cox AD, Der CJ (2002). Farnesyltransferase inhibitors: promises and realities. Current opinion in pharmacology.

[14] Califano R, Landi L, Cappuzzo F (2012). Prognostic and predictive value of K-RAS mutations in non-small cell lung cancer. Drugs.

[15] Bhat M (2015). Targeting the translation machinery in cancer. Nature reviews. Drug discovery.

[16] Colina R (2008). Translational control of the innate immune response through IRF-7. Nature.

[17] Hanahan D, Weinberg RA (2011). Hallmarks of cancer: the next generation. Cell.

[18] Topisirovic I, Sonenberg N (2011). mRNA translation and energy metabolism in cancer: the role of the MAPK and mTORC1 pathways. Cold Spring Harbor symposia on quantitative biology.

[19] Kantarjian HM (2001). Homoharringtonine: history, current research, and future direction. Cancer.

[20] Huang CC (1983). Cytotoxicity and sister chromatid exchanges induced *in vitro* by six anticancer drugs developed in thePeople’s Republic of China. Journal of the National Cancer Institute.

[21] Harringtonine in acute leukemias (1977). Clinical analysis of 31 cases. Chinese medical journal.

[22] Cephalotaxine esters in the treatment of acute leukemia (1976). A preliminary clinical assessment. Chinese medical journal.

[23] Kantarjian HM (1989). Phase II study of low-dose continuous infusion homoharringtonine in refractory acute myelogenous leukemia. Cancer.

[24] Chen R (2011). Homoharringtonine reduced Mcl-1 expression and induced apoptosis in chronic lymphocytic leukemia. Blood.

[25] Gurel G, Blaha G, Moore PB, Steitz TA (2009). U2504 determines the species specificity of the A-site cleft antibiotics: the structures of tiamulin, homoharringtonine, and bruceantin bound to the ribosome. Journal of molecular biology.

[26] Zhou DC, Zittoun R, Marie JP (1995). Homoharringtonine: an effective new natural product in cancer chemotherapy. Bulletin du cancer.

[27] Tujebajeva RM, Graifer DM, Karpova GG, Ajtkhozhina NA (1989). Alkaloid homoharringtonine inhibits polypeptide chain elongation on human ribosomes on the step of peptide bond formation. FEBS letters.

[28] Visani G (1997). Effects of homoharringtonine alone and in combination with alpha interferon and cytosine arabinoside on ‘*in vitro*’ growth and induction of apoptosis in chronic myeloid leukemia and normal hematopoietic progenitors. Leukemia.

[29] Yinjun L, Jie J, Weilai X, Xiangming T (2004). Homoharringtonine mediates myeloid cell apoptosis via upregulation of pro-apoptotic bax and inducing caspase-3-mediated cleavage of poly(ADP-ribose) polymerase (PARP). American journal of hematology.

[30] Tong H, Ren Y, Zhang F, Jin J (2008). Homoharringtonine affects the JAK2-STAT5 signal pathway through alteration of protein tyrosine kinase phosphorylation in acute myeloid leukemia cells. European journal of haematology.

[31] Cao W (2015). Homoharringtonine induces apoptosis and inhibits STAT3 via IL-6/JAK1/STAT3 signal pathway in Gefitinib-resistant lung cancer cells. Scientific reports.

[32] Roux PP (2007). RAS/ERK signaling promotes site-specific ribosomal protein S6 phosphorylation via RSK and stimulates cap-dependent translation. The Journal of biological chemistry.

[33] Jacobson BA (2006). Repression of cap-dependent translation attenuates the transformed phenotype in non-small cell lung cancer both *in vitro* and *in vivo*. Cancer research.

[34] Bracci L, Schiavoni G, Sistigu A, Belardelli F (2014). Immune-based mechanisms of cytotoxic chemotherapy: implications for the design of novel and rationale-based combined treatments against cancer. Cell death and differentiation.

[35] Bustamante Alvarez JG (2015). Advances in immunotherapy for treatment of lung cancer. Cancer biology & medicine.

[36] Topalian SL (2012). Safety, activity, and immune correlates of anti-PD-1 antibody in cancer. The New England journal of medicine.

[37] Brahmer JR (2010). Phase I study of single-agent anti-programmed death-1 (MDX-1106) in refractory solid tumors: safety, clinical activity, pharmacodynamics, and immunologic correlates. Journal of clinical oncology: official journal of the American Society of Clinical Oncology.

[38] Dranoff G (2004). Cytokines in cancer pathogenesis and cancer therapy. *Nature reviews*. Cancer.

[39] Piccirillo CA, Bjur E, Topisirovic I, Sonenberg N, Larsson O (2014). Translational control of immune responses: from transcripts to translatomes. Nature immunology.

[40] Xu, M. *et al*. Regulation of antitumor immune responses by the IL-12 family cytokines, IL-12, IL-23, and IL-27. Clinical & developmental immunology **2010**, 10.1155/2010/832454 (2010). 10.1155/2010/832454 PMC2946577 20885915

[41] Rau FC, Dieter J, Luo Z, Priest SO, Baumgarth N (2009). B7-1/2 (CD80/CD86) direct signaling to B cells enhances IgG secretion. J Immunol.

[42] Teodorovic LS, Riccardi C, Torres RM, Pelanda R (2012). Murine B cell development and antibody responses to model antigens are not impaired in the absence of the TNF receptor GITR. PloS one.

[43] Sahoo NC, Rao KV, Natarajan K (2002). CD80 expression is induced on activated B cells following stimulation by CD86. Scandinavian journal of immunology.

[44] Floyd HS (2005). Conditional expression of the mutant Ki-rasG12C allele results in formation of benign lung adenomas: development of a novel mouse lung tumor model. Carcinogenesis.

[45] Schneider-Poetsch T (2010). Inhibition of eukaryotic translation elongation by cycloheximide and lactimidomycin. Nature chemical biology.

[46] Garreau de Loubresse N (2014). Structural basis for the inhibition of the eukaryotic ribosome. Nature.

[47] Lippitz BE (2013). Cytokine patterns in patients with cancer: a systematic review. The Lancet. Oncology.

[48] Weiner LM, Surana R, Wang S (2010). Monoclonal antibodies: versatile platforms for cancer immunotherapy. Nature reviews. Immunology.

[49] Tsou P, Katayama H, Ostrin EJ, Hanash SM (2016). The Emerging Role of B Cells in Tumor Immunity. Cancer research.

[50] Zhang C (2016). CD5 Binds to Interleukin-6 and Induces a Feed-Forward Loop with the Transcription Factor STAT3 in B Cells to Promote Cancer. Immunity.

[51] de Visser KE, Korets LV, Coussens LM (2005). De novo carcinogenesis promoted by chronic inflammation is B lymphocyte dependent. Cancer cell.

[52] Germain C (2014). Presence of B cells in tertiary lymphoid structures is associated with a protective immunity in patients with lung cancer. American journal of respiratory and critical care medicine.

[53] Lapointe R, Bellemare-Pelletier A, Housseau F, Thibodeau J, Hwu P (2003). CD40-stimulated B lymphocytes pulsed with tumor antigens are effective antigen-presenting cells that can generate specific T cells. Cancer research.

[54] Schultze JL (1997). CD40-activated human B cells: an alternative source of highly efficient antigen presenting cells to generate autologous antigen-specific T cells for adoptive immunotherapy. The Journal of clinical investigation.

[55] de la Iglesia N (2008). Identification of a PTEN-regulated STAT3 brain tumor suppressor pathway. Genes & Development.

[56] Gao SP (2007). Mutations in the EGFR kinase domain mediate STAT3 activation via IL-6 production in human lung adenocarcinomas. The Journal of clinical investigation.

[57] Yu H, Kortylewski M, Pardoll D (2007). Crosstalk between cancer and immune cells: role of STAT3 in the tumour microenvironment. Nature Reviews Immunology.

[58] Matsuyama H (2011). miR-135b mediates NPM-ALK-driven oncogenicity and renders IL-17-producing immunophenotype to anaplastic large cell lymphoma. Blood.

[59] Zheng WP, Flavell RA (1997). The transcription factor GATA-3 is necessary and sufficient for Th2 cytokine gene expression in CD4 T cells. Cell.

[60] Hofman FM (1998). IL-4 regulates differentiation and proliferation of human precursor B cells. Journal of Immunology.

[61] Gysin S, Salt M, Young A, McCormick F (2011). Therapeutic strategies for targeting ras proteins. Genes & cancer.

[62] Yoon YK (2010). KRAS mutant lung cancer cells are differentially responsive to MEK inhibitor due to AKT or STAT3 activation: implication for combinatorial approach. Molecular carcinogenesis.

[63] Puyol M (2010). A synthetic lethal interaction between K-Ras oncogenes and Cdk4 unveils a therapeutic strategy for non-small cell lung carcinoma. Cancer cell.

[64] Scheper GC (2003). The N and C termini of the splice variants of the human mitogen-activated protein kinase-interacting kinase Mnk2 determine activity and localization. Molecular and cellular biology.

[65] Rajasekhar VK (2003). Oncogenic Ras and Akt signaling contribute to glioblastoma formation by differential recruitment of existing mRNAs to polysomes. Molecular cell.

[66] Chu YW (1997). Selection of invasive and metastatic subpopulations from a human lung adenocarcinoma cell line. American journal of respiratory cell and molecular biology.

[67] Weng TY (2014). DNA vaccine elicits an efficient antitumor response by targeting the mutant Kras in a transgenic mouse lung cancer model. Gene therapy.

[68] Carmi Y (2015). Allogeneic IgG combined with dendritic cell stimuli induce antitumour T-cell immunity. Nature.

