# Supplementary material for: Homoharringtonine induced immune alteration for an Efficient Anti-tumor Response in Mouse Models of Non-small Cell Lung Adenocarcinoma Expressing Kras Mutation
Source: Sci Rep. 2018 May 29;8:8216. doi: 10.1038/s41598-018-26454-w (PMC5974086; doi:10.1038/s41598-018-26454-w)
Supplement: Supplementary file 1 — Supplementary Figures [file 41598_2018_26454_MOESM1_ESM.pdf]

Homoharringtonine induced immune alteration for an Efficient Anti-tumor Response in Mouse Models of Non-small Cell Lung Adenocarcinoma Expressing Kras Mutation

Tzu-Yang Weng[1,2], HsuanFranziskaWu[3], Chung-Yen Li[1,4], Yu-Hsuan Hung[4], Yu-Wei Chang[3], Yi-Ling Chen[5], Hui-Ping Hsu[2], Yu-Hung Chen[1, 3], Chih-Yang Wang[4], Jang-Yang Chang[6], Ming-Derg Lai[1,*].

1. Department of Biochemistry and Molecular Biology, College of Medicine, National Cheng Kung University, Tainan, Taiwan.
2. Department of Surgery, National Cheng Kung University Hospital, College of Medicine, National Cheng Kung University, Tainan, Taiwan.
3. Department of Medicine, National Cheng Kung University, Tainan, Taiwan
4. Institute of Basic Medical Sciences, College of Medicine, National Cheng Kung University, Tainan, Taiwan
5. Department of Senior Citizen Services Management, Chia Nan University of Pharmacy and Science, Tainan, Taiwan.
6. National Institute of Cancer Research, National Health Research Institute, Tainan, Taiwan.

* Corresponding author

Ming-Derg Lai, E-mail: a1211207@mail.ncku.edu.tw

Department of Biochemistry and Molecular Biology, College of Medicine, National Cheng Kung University, Tainan, Taiwan.

Tel.: +886-6-2353535#5549, Fax: +886-6-2741694

Supplementary Figures

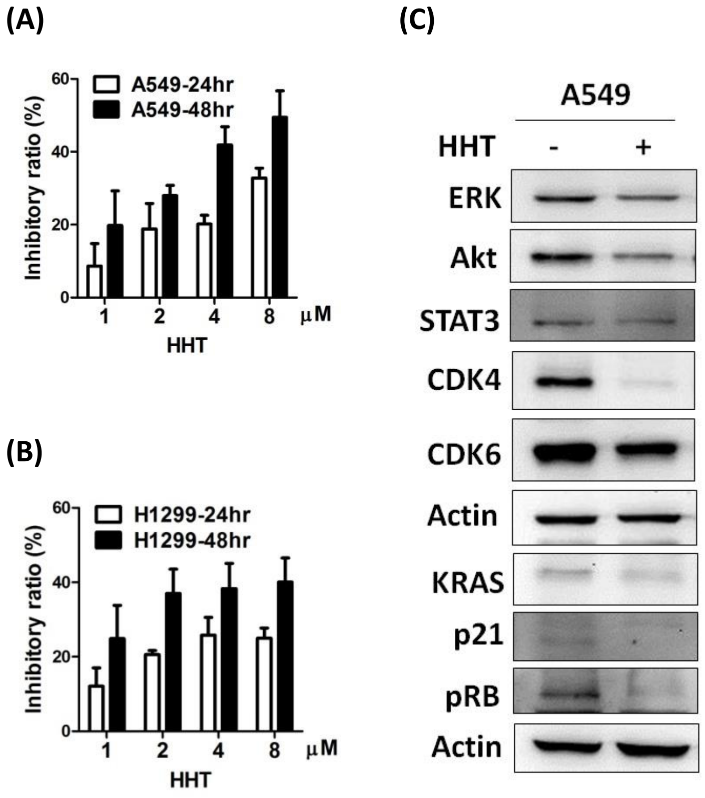

Supplementary Figure 1. Effects of HHT on the inhibition of cell growth in **(A)** the human lung cancer A549 cell line and **(B)** H1299 cell line, as measured by the WST-1 assay. The cells were treated with 1, 2, 4, and 8 μM HHT for 24 and 48 h. The cell viability was measured using a WST-1 assay. Three independent experiments were performed. The protein expression of **(C)** oncogenes and tumor suppressors in A549 cells treated with 2 μM HHT was detected by using Western blot.

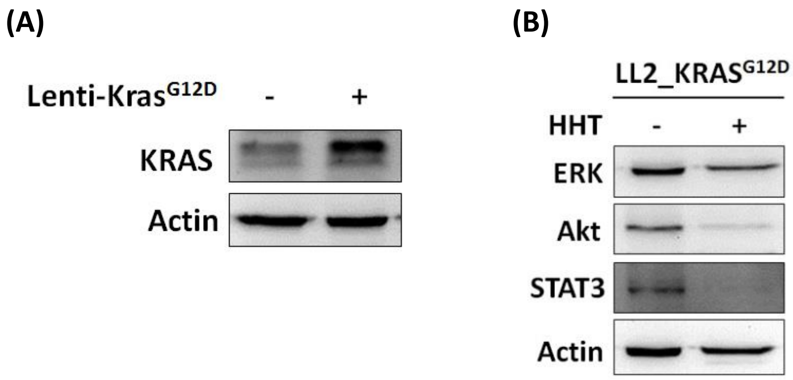

Supplementary Figure 2. (A) LL2 transduced with lentivirus expressed Kras^{G12D}. Kras expression was detected by western blot analysis. **(B)** Protein expression in LL2-expressed Kras^{G12D} cells treated with 2 µM HHT was detected by western blot analysis.

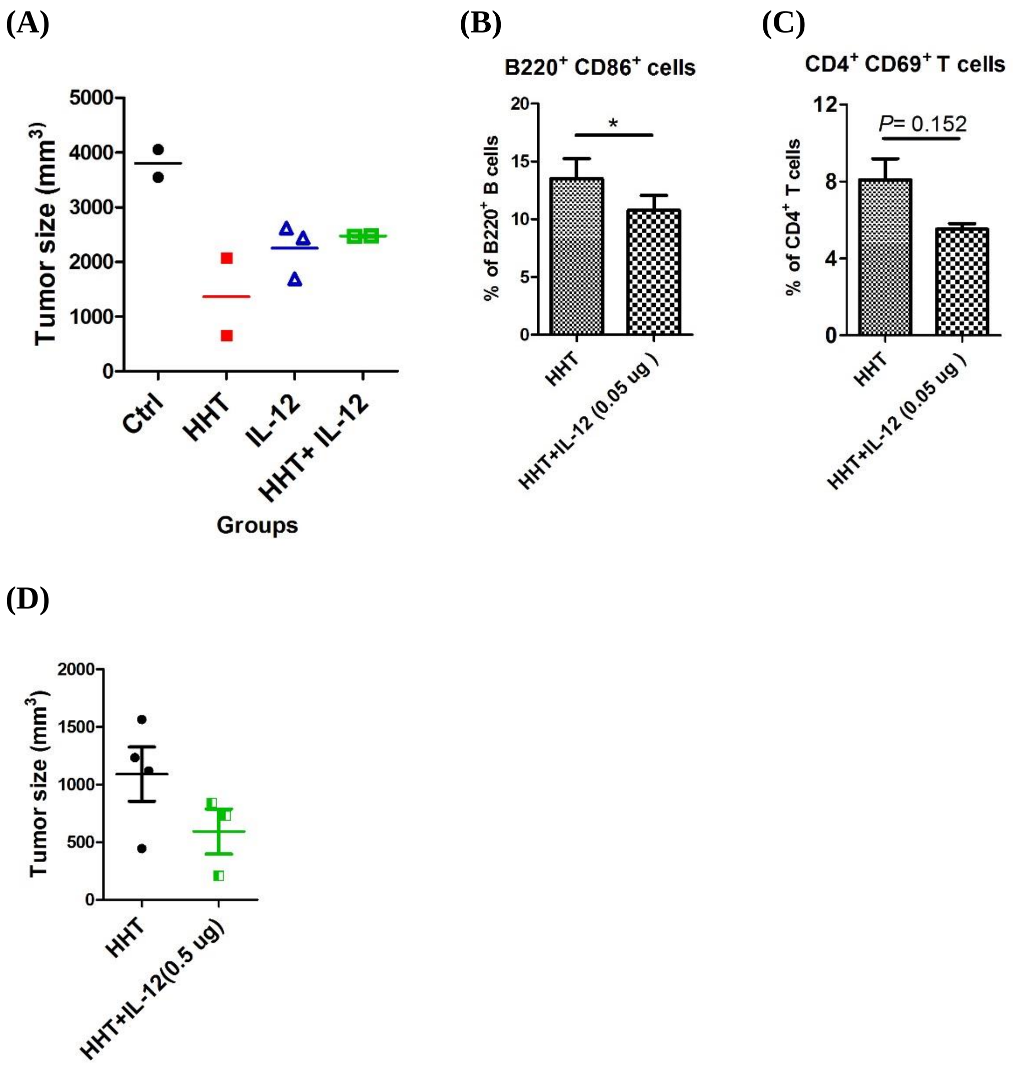

Supplemental Figure 3. (A) Kras^{G12D}-expressing LL2 tumor-bearing mice were treated with HHT (2.5 mg/kg) or IL-12 (0.05 ug/mice), and the tumor volume was measured on day 16. Splenocytes were isolated from Kras^{G12D}-expressing LL2 mice, which were treated with HHT and/or IL-12. **(B)** FACS analysis of B220^{+} CD86^{+} B cells (n = 3 mice per group). **(C)** FACS analysis of CD4^{+} CD69^{+} T cells (n = 2 mice per group). The bar graph represents the average ± SEM. *p< 0.05. **(D) (A)** Kras^{G12D}-expressing LL2 tumor-bearing mice were treated with HHT (2.5 mg/kg), and IL-12 (0.05 ug/mice) or IL-12 (0.5 ug/mice). Tumor volume was measured on day 16.

(A)

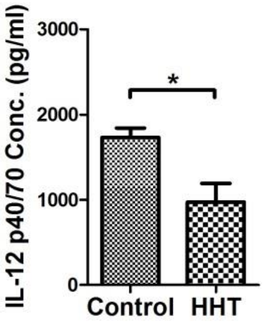

Supplementary Figure 4. IL-12 expression levels of splenocytes isolated from $Kras^{G12C}$-expressing LL2 tumor-bearing mice were measured using an ELISA. The columns and bars represent mean values ± SEM ($n = 3$ per group). $*p < 0.05$.

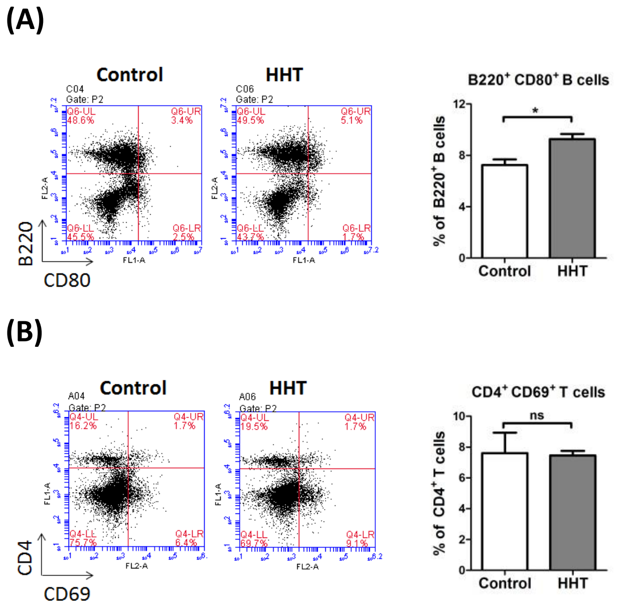

Supplementary Figure 5. Splenocytes were isolated from Kras^{G12C} bi-transgenic mice treated with HHT. **(A)** FACS analysis of B220^{+} CD80^{+} B cells. **(B)** FACS analysis of CD4^{+} CD69^{+} T cells. The bar graph represents the average ± SEM (n = 3 mice per group). *p < 0.05. ns, no statistical difference.

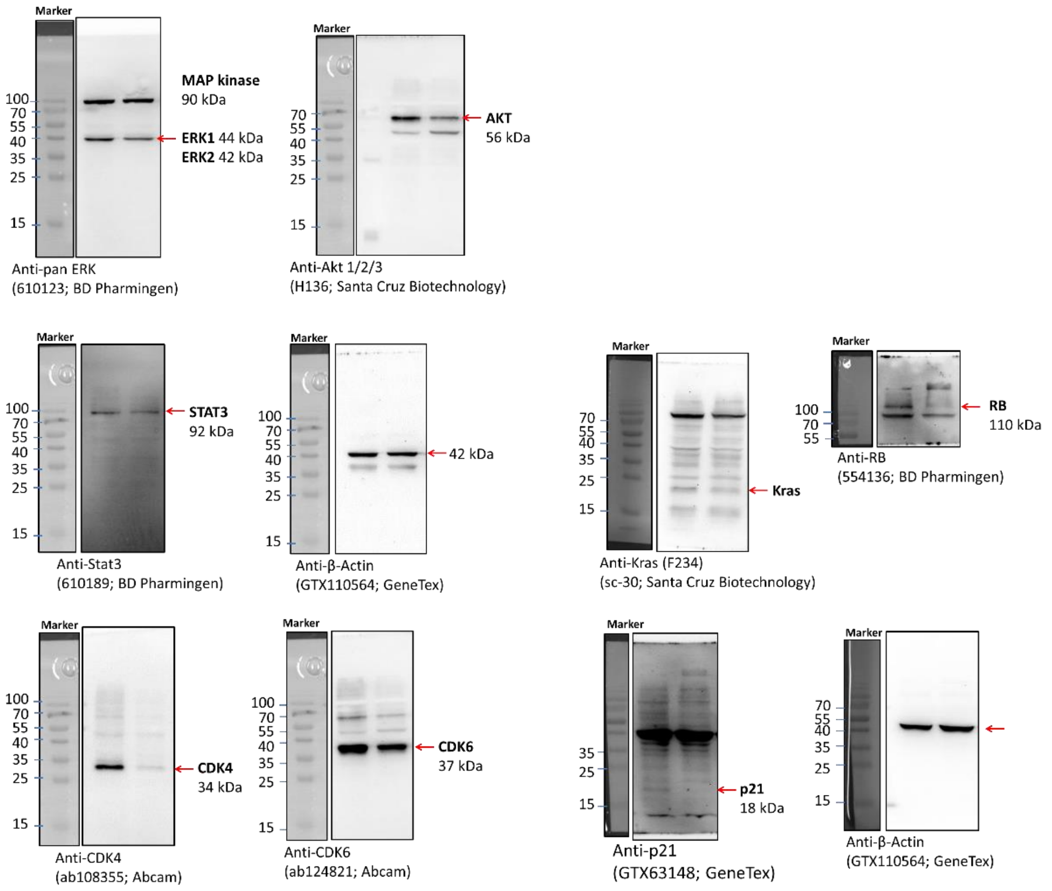

Supplementary Figure 6. The protein expression of ERK, AKT, STAT3, CDK4, CDK6, KRAS, RB, p21 and Actin in A549 cells treated with 2 μM HHT, were detected by using Western blot in Figure S1C.

(A)

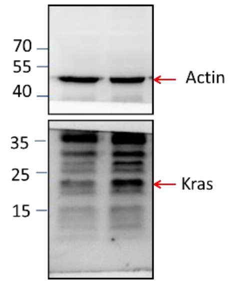

(B)

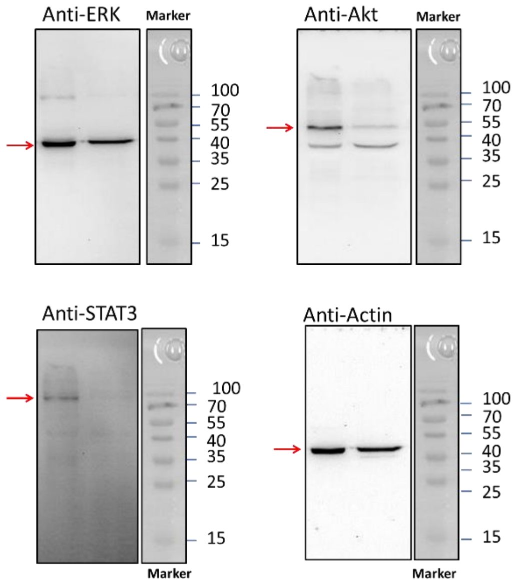

Supplementary Figure 7. (A) The protein expression of LL2 transduced with Kras^{G12D} was detected by using Western blot in Supplementary Figure S2A. **(B)** The protein expression of ERK, AKT, STAT3, and Actin in LL2 cells treated with 2 μM HHT was detected by using Western blot in Supplementary Figure S2B.

**Primer**	**Sequences (5'-3')**
IL12p40 forward	TGC TGG TGT CTC CAC TCA TGG C
IL12p40 reverse	TTT CAG TGG ACC AAA TTC CAT T
IL-4 forward	GAA TGT ACC AGG AGC CAT ATC
IL-4 reverse	CTC AGT ACT ACG AGT AAT CCA
IFN-γ forward	AAC GCT ACA CAC TGC ATC TTG G
IFN-γ reverse	CAA GAC TTC AAA GAG TCT GAG G
IL-10 forward	CCA GTT TTA CCT GGT AGA AGT GAT G
IL-10 reverse	TGT CTA GGT CCT GGA GTC CAG CAG ACT CAA
TNF-α forward	CCC CAA AGG GAT GAG AAG TT
TNF-α reverse	CAC TTG GTG GTT TGC TAC GA
HPRT forward	GTT GGA TAC AGG CCA GAC TTT GTT G
HPRT reverse	GAT TCA ACT TGC GCT CAT CTT AGG C

Supplementary Table S1. Primers for real time-PCR.
